# Diabetes prediction using machine learning and explainable AI techniques

**DOI:** 10.1049/htl2.12039

**Published:** 2022-12-14

**Authors:** Isfafuzzaman Tasin, Tansin Ullah Nabil, Sanjida Islam, Riasat Khan

**Affiliations:** ^1^ Electrical and Computer Engineering North South University Dhaka Bangladesh

**Keywords:** AdaBoost, android Application, decision tree, diabetes, K‐nearest neighbour, random forest, support vector machine

## Abstract

Globally, diabetes affects 537 million people, making it the deadliest and the most common non‐communicable disease. Many factors can cause a person to get affected by diabetes, like excessive body weight, abnormal cholesterol level, family history, physical inactivity, bad food habit etc. Increased urination is one of the most common symptoms of this disease. People with diabetes for a long time can get several complications like heart disorder, kidney disease, nerve damage, diabetic retinopathy etc. But its risk can be reduced if it is predicted early. In this paper, an automatic diabetes prediction system has been developed using a private dataset of female patients in Bangladesh and various machine learning techniques. The authors used the Pima Indian diabetes dataset and collected additional samples from 203 individuals from a local textile factory in Bangladesh. Feature selection algorithm mutual information has been applied in this work. A semi‐supervised model with extreme gradient boosting has been utilized to predict the insulin features of the private dataset. SMOTE and ADASYN approaches have been employed to manage the class imbalance problem. The authors used machine learning classification methods, that is, decision tree, SVM, Random Forest, Logistic Regression, KNN, and various ensemble techniques, to determine which algorithm produces the best prediction results. After training on and testing all the classification models, the proposed system provided the best result in the XGBoost classifier with the ADASYN approach with 81% accuracy, 0.81 F1 coefficient and AUC of 0.84. Furthermore, the domain adaptation method has been implemented to demonstrate the versatility of the proposed system. The explainable AI approach with LIME and SHAP frameworks is implemented to understand how the model predicts the final results. Finally, a website framework and an Android smartphone application have been developed to input various features and predict diabetes instantaneously. The private dataset of female Bangladeshi patients and programming codes are available at the following link: https://github.com/tansin-nabil/Diabetes-Prediction-Using-Machine-Learning.

## Graphical abstract

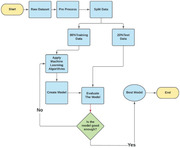

## 1 INTRODUCTION

Diabetes is a chronic disease that directly affects the pancreas, and the body is incapable of producing insulin [22]. Insulin is mainly responsible for maintaining the blood glucose level. Many factors, such as excessive body weight, physical inactivity, high blood pressure, and abnormal cholesterol level, can cause a person get affected by diabetes [23]. It can cause many complications, but an increase in urination is one of the most common ones [24]. It can damage the skin, nerves, and eyes, and if not treated early, diabetes can cause kidney failure and diabetic retinopathy ocular disease. According to IDF (International Diabetes Federation) statistics, 537 million people had diabetes around the world in 2021 [1]. In Bangladesh, approximately 7.10 million people had suffered from this disease, according to 2019 statistics [2].

Early and accurate diagnosis of diabetes mellitus, especially during its initial development, is challenging for medical professionals. Artificial intelligence and machine learning techniques, providing a reference, can help them gain preliminary knowledge about this disease and reduce their workload accordingly. Significant numbers of research have been performed to predict diabetes automatically using machine learning and ensemble techniques. Most of these works employed the open‐source Pima Indian dataset [6]. Some of these articles on automatic diabetes prediction employing the Pima Indian dataset are briefly discussed in the following paragraphs. For instance, Kumar et al. [4] used the random forest algorithm to design a system that can predict diabetes quickly and accurately. The dataset used in this work was collected from the UCI learning repository. First, the authors used conventional data preprocessing techniques, including data cleaning, integration, and reduction. The accuracy level was 90% using the random forest algorithm, which is much higher when compared to other algorithms. In a recent paper [5], Mohan and Jain used the SVM algorithm to analyze and predict diabetes with the help of the Pima Indian Diabetes Dataset. This work used four types of kernels, linear, polynomial, RBF, and sigmoid, to predict diabetes in the machine learning platform. The authors obtained diverse accuracies in different kernels, ranging between 0.69 and 0.82. The SVM technique with radial basis kernel function obtained the highest accuracy of 0.82. Goyal and his team [9] created a smart home health monitoring scheme to detect diabetes. The authors also employed the Pima Indian dataset for their research. For predicting blood pressure status, they used conditional decision making and for predicting diabetes, they used SVM, KNN, and decision tree. Among these models, SVM worked better as they got 75% accuracy which is better than other classifier algorithms. Hassan et al. [10] attempted to predict diabetes using different ensemble method‐based machine learning algorithms and the Pima Indian dataset. The authors considered AUC (area under the ROC curve) as their accuracy measure. Finally, the proposed ensemble classifier accomplished an AUC value of 0.95. Jackins et al. [17] proposed a multi‐disease prediction system, including diabetes using machine learning techniques and the Pima Indian dataset. According to the authors, the Naive Bayes performed better than the random forest technique with accuracy increments of 0.43%. Mounika et al. [19] anticipated diabetes probabilities using machine learning techniques. This work employed the public Pima Indian dataset and multiple machine learning frameworks. Kumari et al. [21] attempted to apply a soft voting classifier‐based ensemble approach for diabetes prediction. The proposed soft voting classifier attained the overall highest accuracy and F1 score of 0.791 and 0.716, respectively. Prabhu and Selvabharathi [3] used the open‐source Pima Indian diabetes dataset for predicting diabetes using the deep belief network model. The authors constructed the model in three phases, that is, data preprocessing using min–max normalization, constructing the network model, and fine‐tuning the test dataset to remove any partiality using NN‐FF classification. Finally, the authors have done all the implementation and simulation of the model using MATLAB. The authors reported an F1 score of 0.808, finding the best performance metric compared with the other classification methods.

Some of these works employed custom datasets or a combination of different datasets. In [14], the authors proposed a type 2 diabetes early prediction system using machine learning approaches. The authors employed a private dataset with more than 253,000 volunteer data from a local hospital in Korea for 6 years. Synthetic oversampling, SMOTE, and undersampling algorithms are applied to deal with the data imbalance problem. Various machine learning approaches are used to anticipate this disease for the following year from the past year's patients’ data. Both the random forest and SVM classifiers achieved the highest F1 score of 74%. Pranto et al. [12] utilized Pima Indian and a private dataset from a local hospital in Bangladesh to design an automatic diabetes prediction system. This work trained several machine learning techniques on the Pima Indian dataset. KNN and decision tree models achieved 81.2% and 79.2% accuracies on the private dataset, respectively. Olisah et al. [15] implemented diabetes mellitus forecasting using advanced feature selection and machine learning models. The authors employed two open‐source datasets, that is, Pima Indian and LMCH Iraqi databases. A polynomial regression‐based preprocessing technique was used for predicting the missing samples. Hyperparameter tuning has been performed for the random forest, decision tree, and deep neural network frameworks. The proposed DNN technique with the optimized hyperparameters accomplished the highest accuracies of 0.972 and 0.973 for the Pima and LMCH datasets, respectively.

The applied machine learning models have been deployed into a website or smartphone application in some of the articles. In one study, the authors [16] designed a website for the automatic prediction of diabetes. This work employed two open‐source datasets and various popular machine learning approaches. The decision tree and random forest classifiers obtained the highest performance for this work with an accuracy of 0.968. Ramesh et al. [18] designed a remote and automatic system for diabetes forecasting with the Pima Indian dataset. The authors employed different data preprocessing techniques, that is, feature scaling, feature selection, and SMOTE. SVM with RBF kernel attained a maximum accuracy of 83.2%. The proposed ML framework is employed in an Android application.

We draw the conclusion that researchers have successfully combined multiple machine learning algorithms with diverse data preprocessing approaches for automatic diabetes detection by reviewing the relevant articles. Most of the works focused on a single accuracy measure, used the open‐source Pima Indian dataset, and did not develop the explicability of the prediction of the machine learning frameworks. These reasons have motivated us to evaluate our proposed prediction system based on accuracy, precision, recall, and F1 score, utilize more custom data to merge with the existing dataset, and apply an explainable AI technique.

In this paper, we have employed machine learning and explainable AI techniques to detect diabetes. Along with a private dataset from employees of a local textile industry in Bangladesh, we used the Pima Indian dataset in this paper [6]. As there were many missing values in some attributes, we replaced them with the mean value of each feature. We have used the holdout validation technique to split the data. In this research paper, we have applied various machine learning‐based classification algorithms, that is, decision tree, logistic regression, KNN, random forest, SVM, and ensemble techniques. Next, the performance of these classifiers has been evaluated in terms of precision, recall, and F1 measure. Finally, the best classifier has been selected as the final model to deploy into an Android smartphone application.

This paper implements diabetes mellitus prediction through machine learning. The significant contribution of this work is as follows:

- A significant contribution of this work is to present a unique dataset of diabetes mellitus containing 203 samples. This private dataset has been obtained from female employees of Rownak Textile Mills Ltd, Dhaka, Bangladesh, referred to as the ‘RTML dataset’ in this paper. We have collected six features from 203 individuals, that is, pregnancy, glucose, blood pressure, skin thickness, BMI, age, and final outcome of diabetes.
- Another contribution of this work is to keep similarities with the feature of the Pima Indian dataset. The missing insulin feature of the RTML dataset was predicted using a semi‐supervised technique.
- SMOTE and ADASYN techniques are implemented to minimize the class imbalance issue. Hyperparameter tuning has also been performed in this work.
- Explainable AI technique with SHAP and LIME libraries is implemented to understand how the model predicts the decision. This approach helps to interpret what features play the most crucial role in terms of prediction.
- A website and an Android application have been developed with the finalized best‐performed model of this research work to make instantaneous predictions with real‐time data.

The novelty of this work is to implement an automatic diabetes prediction website and Android application for a private dataset of female Bangladeshi patients using machine learning and ensemble techniques.

The following paragraph is a breakdown of the paper's structure. The proposed automatic diabetes prediction system has been discussed and illustrated in Section 2 with suitable figures and flowcharts. The final results of the research are presented in Section 3. Finally, Section 4 concludes the paper with some recommendations for future improvements.

## 2 PROPOSED SYSTEM

**FIGURE 1:**
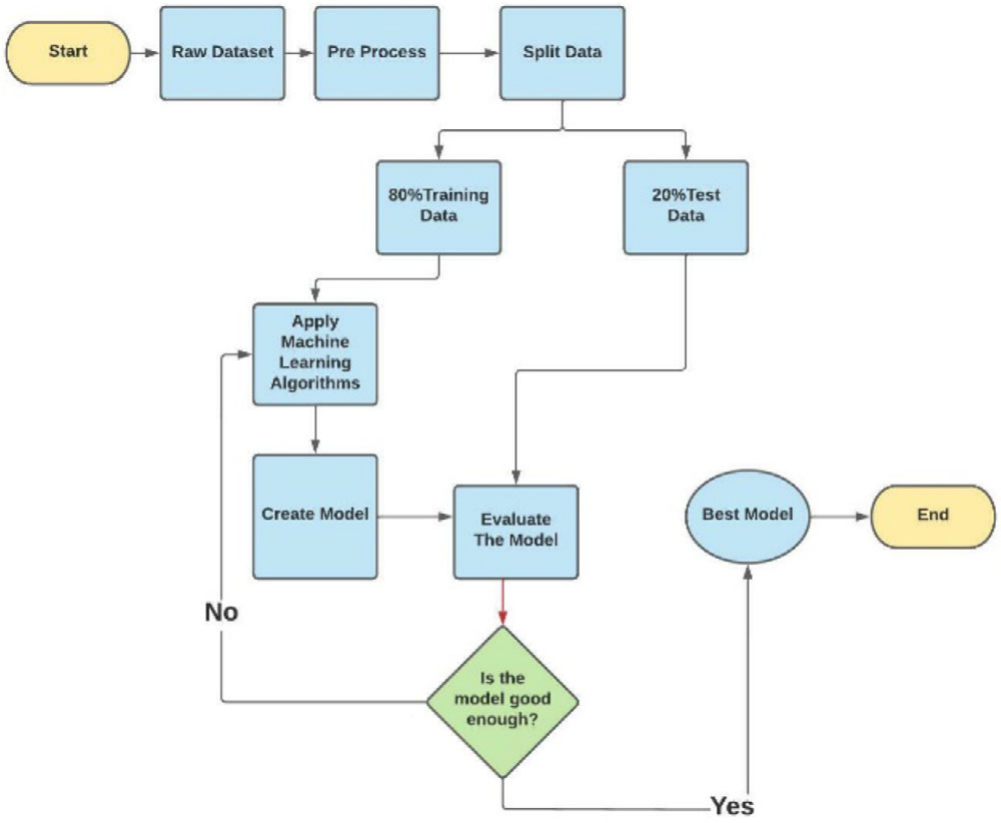
Working sequences of the proposed diabetes prediction system

This section describes the working procedures and implementation of various machine learning techniques to design the proposed automatic diabetes prediction system. Figure 1 shows the different stages of this research work. First, the dataset was collected and preprocessed to remove the necessary discrepancies from the dataset, for example, replacing null instances with mean values, dealing with imbalanced class issues etc. Then the dataset was separated into the training set and test set using the holdout validation technique. Next, different classification algorithms were applied to find the best classification algorithm for this dataset. Finally, the best‐performed prediction model is deployed into the proposed website and smartphone application framework.

### 2.1 Dataset

The Pima Indian dataset is an open‐source dataset [6] that is publicly available for machine learning classification, which has been used in this work along with a private dataset. It contains 768 patients’ data, and 268 of them have developed diabetes.

**FIGURE 2:**
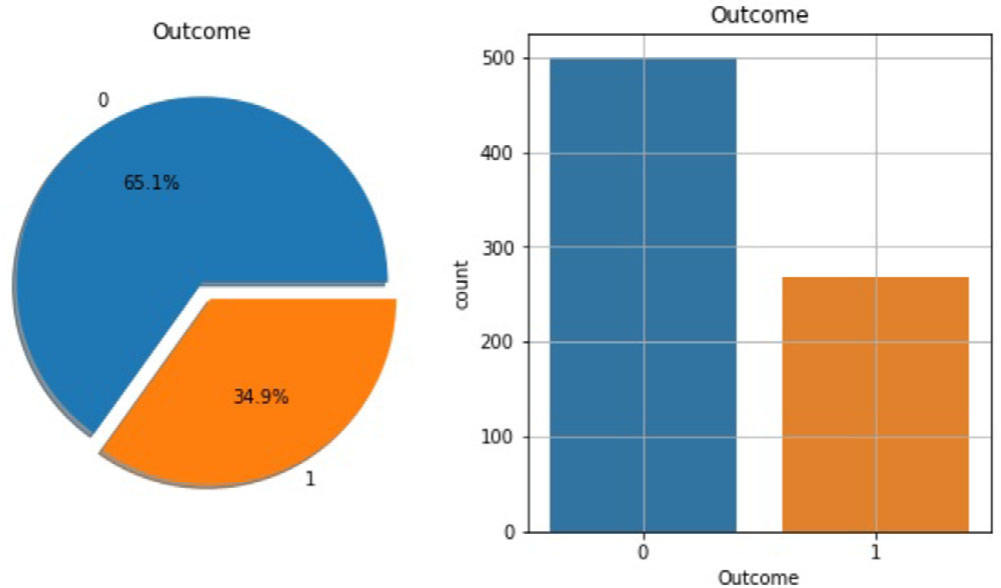
Percentage of people having diabetes in the Pima Indian dataset

Figure 2 shows the ratio of people having diabetes in the Pima Indian dataset. Table 1 demonstrates the eight features of the open‐source Piman Indian dataset.

**TABLE 1:** Features of the Pima Indian Dataset

**Pregnancies**	**Skin thickness**	**Diabetes pedigree function**
Glucose	Insulin	Age
Blood pressure	BMI	

RTML private dataset: A significant contribution of this work is to present a private dataset from Rownak Textile Mills Ltd, Dhaka, Bangladesh, referred to as RTML, to the scientific community. Following a brief explanation of the study to the female volunteers, they voluntarily agreed to participate in the study. This dataset comprises six features, that is, pregnancy, glucose, blood pressure, skin thickness, BMI, age, and outcome of diabetes from 203 female individuals aged between 18 and 77. In this work, blood glucose was measured by the GlucoLeader Enhance blood sugar meter. The blood pressure and skin thickness of the participants were obtained by OMRON HEM‐7156T and digital LCD body fat caliper machines, respectively. Table 2 illustrates distinct features of the private RTML dataset with their minimum, maximum, and average values.

**TABLE 2:** Features of the RTML private dataset

**Features**	**Minimum**	**Maximum**	**Average**
Pregnancies	0	8	1.61
Glucose (mg/dL)	52.2	274	109.39
Blood pressure (mm Hg)	5.9	115	71.09
Skin thickness (mm)	2.9	23.3	10.78
BMI (kg/m^2^)	2.61	41.62	22.69
Age (years)	17	77	27.02

### 2.2 Dataset preprocessing

In the merged dataset, we discovered a few exceptional zero values. For example, skin thickness and Body Mass Index (BMI) cannot be zero. The zero value has been replaced by its corresponding mean value. The training and test dataset has been separated using the holdout validation technique, where 80% is the training data and 20% is the test data.

Mutual Information: Mutual information attempts to measure the interdependence of variables. It produces information gain, and its higher values indicate greater dependency [8].

**FIGURE 3:**
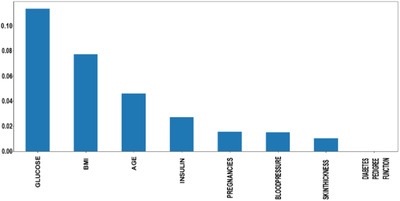
Feature importance hierarchy

Figure 3 shows the mutual information of various features, that is, the importance of each attribute of this dataset. For example, according to this figure, the diabetes pedigree function seems less important according to this mutual information technique.

Semi‐supervised learning: A combined dataset has been used in this work by incorporating the open‐source Pima Indian and private RTML datasets. According to Table 2, the RTML dataset does not contain the insulin feature, which is predicted using a semi‐supervised approach. Before merging the collected dataset with the Pima Indian dataset, a model was created using the extreme gradient boosting technique (XGB regressor). Various regression and ensemble learning techniques have been successfully used in many works to predict missing values [25, 26]. An extensive investigation has been performed while choosing the best‐performed regressor technique to predict the insulin feature of the RTML dataset from the Pima Indian dataset. As the actual value of the insulin was not available in the RTML dataset, the Pima Indian dataset was initially used to select the best regression model. First, the Pima Indian dataset was divided into an 8:2 ratio and three supervised regression models, extreme gradient boosting technique (XGB), support vector regression (SVR), and Gaussian process regression (GPR), have been employed to predict the selected outcome, that is, insulin of the validation samples of the Pima Indian dataset. Next, we computed the root mean square error (RMSE) of various regression frameworks as

(1) $$RMSE\;=\sqrt{\frac{\sum_{i=1}^{N}{\left(Predicted_{i}-Actual_{i}\right)}^{2}}{N}}\;$$

where *N* denotes the total number of validation samples of the Pima Indian dataset.

**FIGURE 4:**
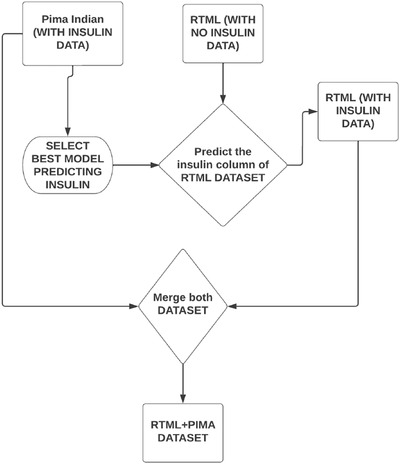
Working steps of predicting insulin of the RTML dataset

According to Table 3, the XGB technique exhibits the lowest RMSE of insulin on the Pima Indian dataset. Therefore, this model has been used to predict the missing insulin column of the collected RTML dataset from the Pima Indian dataset. The working steps of predicting insulin in the RTML dataset have been illustrated in Figure 4.

**TABLE 3:** RMSE of various regression models on the Pima Indian dataset

**Regression model**	**RMSE**
XGB	0.36
SVR	0.45
GPR	0.43

Merged dataset: After the semi‐supervised approach, we predicted the insulin feature and merged the RTML dataset with the Pima Indian dataset. The merged dataset contained 877 data with all the features, excluding the diabetes pedigree function, as it was the least important feature according to mutual information.

SMOTE and ADASYN for class imbalance: The merged dataset used in this work comprises the imbalance problem with 302 and 669 diabetes and non‐diabetes samples, respectively. To take care of this problem, the SMOTE and ADASYN techniques have been applied to the training dataset, leaving the testing data unaffected. Adaptive Synthetic Sampling, known as ADASYN, is a synthetic data generation technique with the characteristics of not duplicating minority samples and generating more data for ‘harder to learn’ examples [13]. As a result, the minority class will be sampled to the same extent as the majority class.

Min–Max normalization: In this research, we used the min–max normalization technique. The data has been scaled to the same range using the following equation:

(2) $$X_{\mathrm{scaled}}=\frac{X-X_{\min}}{X_{\max}-X_{\min}}\;$$

where *X*_max_ and *X*_min_ denote maximum and minimum values in the individual feature column, respectively.

### 2.3 Machine learning classifiers

In this work, various machine learning and ensemble techniques have been employed to implement the automatic diabetes prediction system, briefly discussed below. GridSearchCV framework has been employed in this research to find the optimal values of different hyperparameters for all the machine learning models to prevent overfitting.

Decision tree: A decision tree represents the learning function provided by a set of rules. The decision tree learning technique performs a method for approximating discrete‐valued target functions. Gini or entropy [7] are used to determine information gain, and each node is chosen based on these coefficients, which are expressed as

(3) $$\mathrm{Gin}i_{i}=\;1-\sum_{k\;=\;1}^{n}{\left(p_{i,k}\right)}^{2}$$

(4) $$Entropy\;=\sum_{i=1}^{n}-p_{i}\log_{2}p_{i}$$

In (3) and (4), *n* represents the number of distinct class values. We observed that max depth = 2, minimum samples leaf = 50, and ‘Gini’ impurity metrics work well in the employed dataset in this work using the GridSearchCV hyperparameter tuning.

KNN classifier: A discrete‐valued function can be approximated by *K* number of nearest classifiers [8]. To categorize, it creates a plane with the available training points and calculates the distance between the query and trained points. It determines the *K* number of neighbours (depending on the dataset) and classifies them using majority voting. In our research, we used *K* = 5 for the binary classification.

Random forest: Random forest is a machine learning system that averages the predictions of several decision trees. As a result, the random forest can be considered an ensemble learning model [7]. In this research, we have applied random forest with estimators = 400, minimum samples leaf = 5, and ‘Gini’ impurity metrics utilizing hyperparameter tuning.

Support vector machine: SVM performs supervised classification by choosing the best hyperplane [11]. In this study, we experimented with various SVM kernels in the training set. Finally, we discovered the SVM with a linear kernel, parameters *C* = 10 and gamma = 1, produces the best results in this dataset.

Logistic regression: Logistic regression can be used to predict a binary class. To predict the outcome, it fits an ‘S’ shaped function [8]. The hyperparameter optimization technique obtained the maximum number of iterations for the convergence of the logistic regression model to be 150.

AdaBoost: AdaBoost is an ensemble technique. This classifier initially works on the original dataset, then fits repeated copies of the classifier to the same dataset. This framework adjusts the weights of improperly classified instances so that successive classifiers focus more on difficult circumstances. We have applied AdaBoost with estimator = 50 and learning rate = 0.10 in this work.

XGBoost: XGBoost is an ensemble machine learning technique based on decision trees that employ a gradient boosting approach [20]. The parameters used for the proposed XGBoost classifier are as follows: estimators’ maximum depth = 4 and ‘binary logistic’ objective function.

Voting classifier: It is an ensemble technique to improve the classification by voting [7]. This paper implements a voting classifier that selects the majority class predicted by each classifier with a ‘soft’ voting hyperparameter.

Bagging: Bagging classifiers are ensemble classifiers that fit base classifiers to random subsets of the original dataset and then aggregate their individual predictions voting to generate a final classification [8]. In the implemented bagging classifier, base estimators = 500, maximum number of samples = 100, and out‐of‐bag score = ‘True’ are used as various hyperparameters.

### 2.4 Deployment of the prediction system

The proposed machine learning‐based diabetes prediction system has been deployed into a website and smartphone application framework to work instantaneously on real data.

**FIGURE 5:**
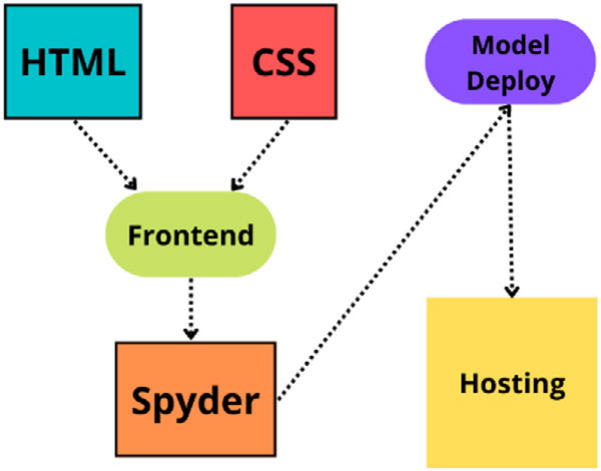
Development of the web application

Web application: We have used HTML and CSS for the frontend part of the proposed website. After that, we finalized the machine learning model XGBoost with ADASYN, as it provided the best performance. The model deployment has been done with Spyder, a Python environment platform that works with Anaconda. Figure 5 shows the illustration of the website application development process.

**FIGURE 6:**
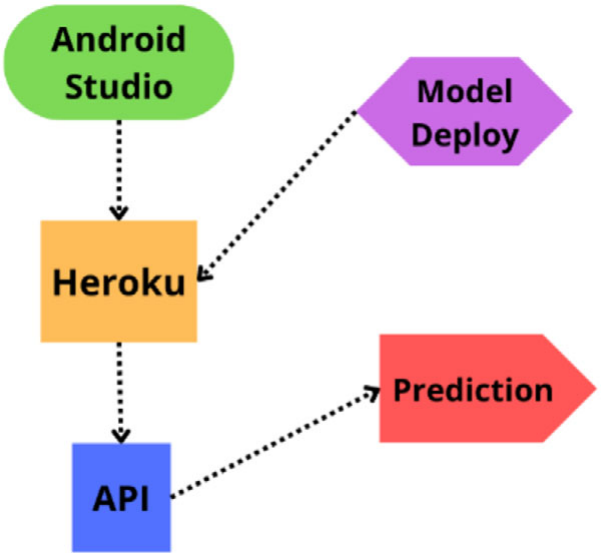
Working sequences of the proposed android application development

Android smartphone application: To demonstrate the automatic diabetes forecasting system in real time, we also designed an Android smartphone application to test its performance. Android Studio is used for the frontend part of this application. We employed Java as the necessary coding language. After that, the model has been implemented in Android Studio using the pickle package. While developing the API, we used Heroku to host our model on the corresponding hosting server. Figure 6 demonstrates the necessary steps in developing the proposed Android application.

## 3 RESULTS AND DISCUSSION

This section presents the results and discussion of the proposed automatic diabetes prediction system. First, the performance of various machine learning techniques is discussed. Next, the implemented website framework and Android smartphone application are demonstrated. We used precision, recall, F1 score, AUC, and classification accuracy to evaluate various ML models. Equations of these metrics are expressed as

(5) $$Precision\;=\frac{TP}{TP+FP}\;$$

(6) $$Recall\;=\frac{TP}{TP+FN}\;$$

(7) $$F1\;score\;=\frac{2\times Recall\times Precision}{Recall+Precision}\;$$

where TP denotes the model is predicting positive, and the result is also positive. FP indicates the positive prediction of the model, but the result is negative. TN expresses the model is predicting negative, and the result is also negative. FN indicates the model predicts negative, but the result is positive. In this work, the holdout validation approach with a stratified 8:2 train‐test split has been used for all the machine learning models.

Table 4 compares different performance metrics of various classifiers for the merged dataset with SMOTE synthetic oversampling technique. According to this table, the bagging classifier achieved the best overall performance with 79% accuracy and 0.79 and 0.87 F1 score and AUC, respectively.

**TABLE 4:** Performance metrics of various classifiers with SMOTE technique in the merged dataset

**Classifier**	**Precision**	**Recall**	**F1 Score**	**Accuracy**	**AUC**
Logistic regression	0.78	0.77	0.77	77%	0.88
KNN	0.78	0.76	0.76	76%	0.85
Random forest	0.78	0.78	0.78	78%	0.87
Decision tree	0.75	0.73	0.73	73%	0.75
**Bagging**	**0.80**	**0.79**	**0.79**	**79%**	**0.87**
Adaboost	0.79	0.78	0.78	78%	0.85
XGboost	0.78	0.78	0.78	78%	0.84
Voting	0.79	0.79	0.79	79%	0.86
SVM	0.78	0.75	0.76	75%	0.87

Table 5 shows various performance metrics of all the classifiers using the ADASYN approach in the merged datasets. According to Table 4, the XGBoost framework performed better than other classifiers with 81% accuracy and 0.84 AUC. Conversely, the decision tree approach achieved the lowest accuracy and F1 score.

**TABLE 5:** Performance metrics of various classifiers using adasyn in the merged dataset

**Classifier**	**Precision**	**Recall**	**F1 Score**	**Accuracy**	**Auc**
Logistic regression	0.76	0.75	0.75	75%	0.84
KNN	0.76	0.73	0.73	73%	0.82
Random forest	0.76	0.76	0.76	76%	0.84
Decision tree	0.81	0.72	0.72	72%	0.78
Bagging	0.80	0.79	0.79	79%	0.84
AdaBoost	0.75	0.76	0.76	76%	0.84
**XGBoost**	**0.81**	**0.81**	**0.81**	**81%**	**0.84**
Voting	0.77	0.77	0.77	77%	0.84
SVM	0.78	0.78	0.77	78%	0.83

Next, the domain adaptation approach has been applied where the machine learning model is trained and evaluated on different samples, that is, source and target datasets, respectively. In this work, initially, the automatic diabetes prediction model is trained on the open‐source Pima Indian dataset with a larger size. Finally, the model is evaluated on the private RTML dataset with a much smaller dimension. Table 6 demonstrates the performance metrics for the private dataset. It is interesting to note that the XGBoost with ADASYN framework has been applied in the training dataset in this case.

**TABLE 6:** Performance metrics for the private dataset (domain adaptation technique)

**Precision**	**Recall**	**F1 score**	**Accuracy**
0.95	0.96	0.95	96%

**FIGURE 7:**
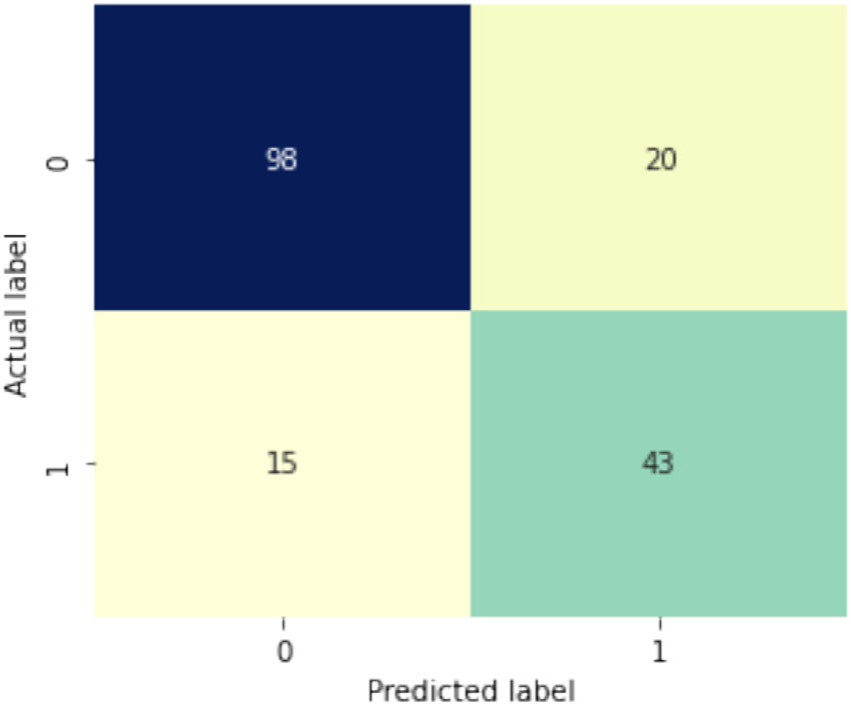
Confusion matrix for XGBoost with ADASYN technique

Figure 7 depicts the confusion matrix for XGBoost with ADASYN. According to this figure, the XGBoost technique correctly classified 141 instances with TP = 43 and TN = 98.

**FIGURE 8:**
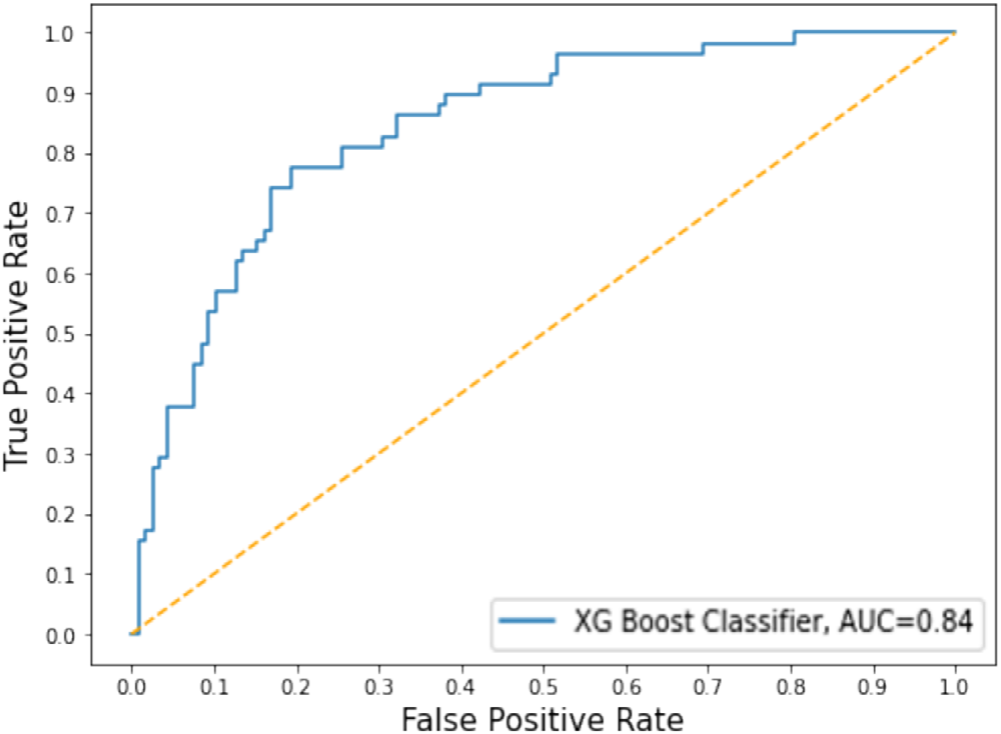
ROC curve and AUC value for the XGBoost with ADASYN

The ROC curve of the XGBoost with the ADASYN approach has been illustrated in Figure 8. This figure shows the AUC value of XGBoost is 0.84.

**FIGURE 9:**
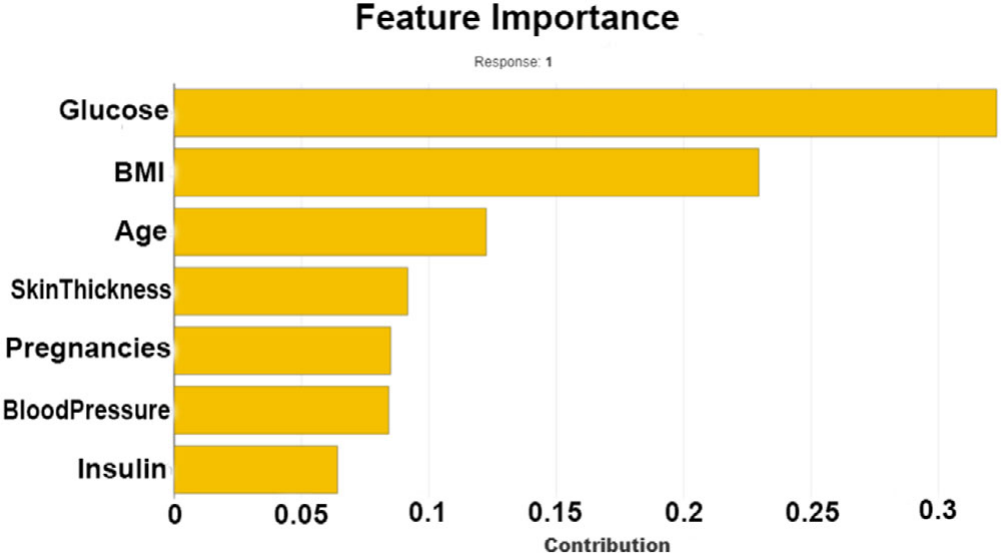
Explainable AI interpretation of feature importance of XGBoost with ADASYN

Next, explainable AI techniques with SHAP and LIME frameworks are implemented to understand how the model predicts the decision. Figure 9 shows the XGBoost with ADASYN feature importance with the help of explainable AI, SHAP library.

**FIGURE 10:**
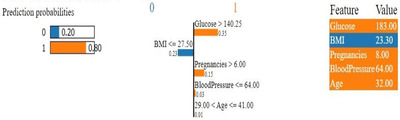
LIME explainable AI prediction interpretation

Figure 10 illustrates an interpretation of the XGBoost model implemented by the LIME explainable AI method. According to this figure, the model predicts diabetes correctly for this specific person with 80% confidence. The ML model predicts this class as the person has a glucose level of more than 140.25 and involves pregnancies of more than 6.

**FIGURE 11:**
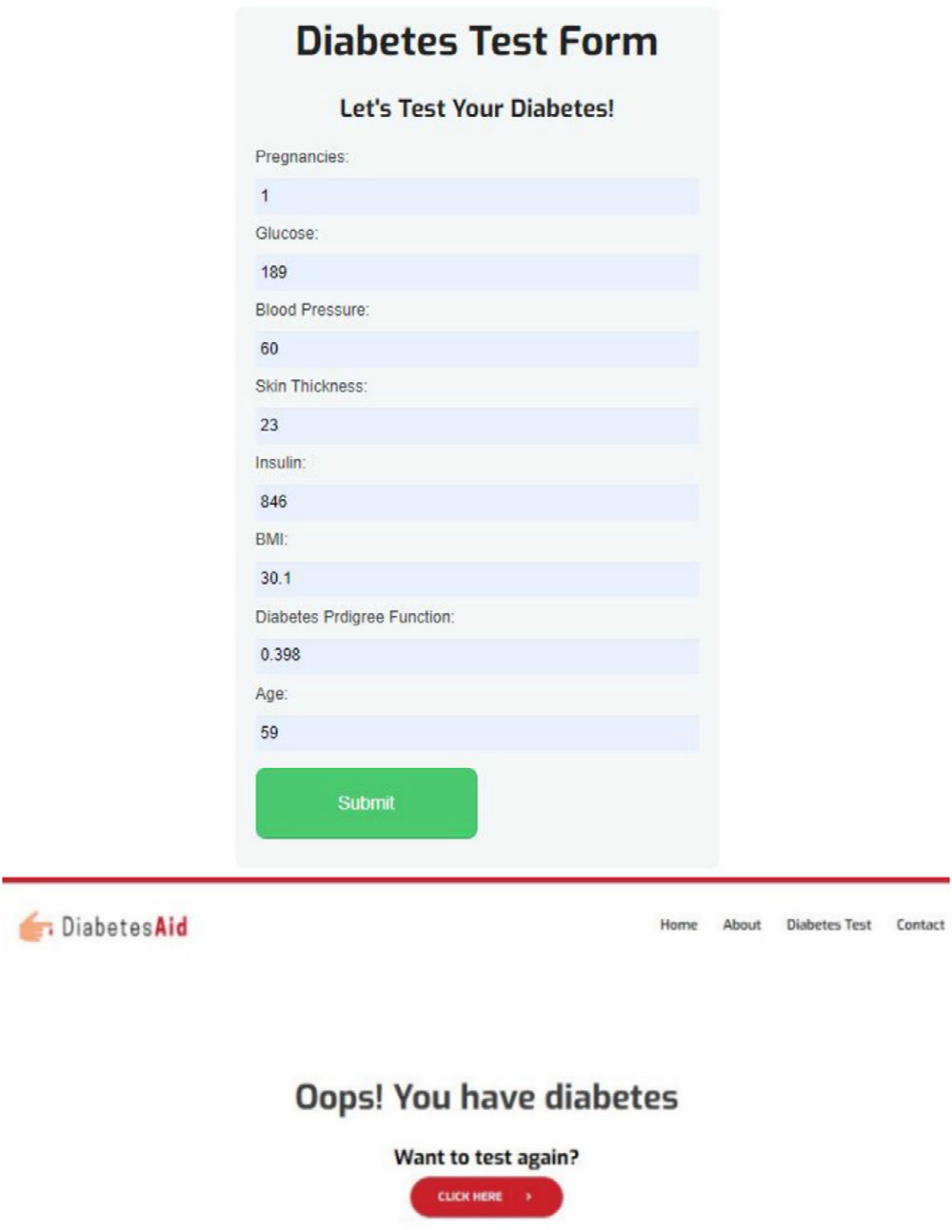
Instantaneous diabetes prediction by the designed web application

Finally, the proposed automatic diabetes prediction system has been deployed into a website and Android smartphone application employing the XGBoost machine learning framework with ADASYN. Figure 11 shows an instantaneous diabetes prediction by the designed web application with real data.

**FIGURE 12:**
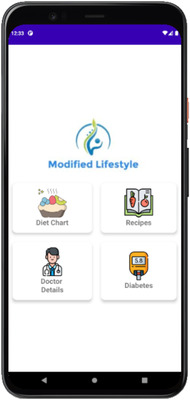
Home screen of the proposed android application

**FIGURE 13:**
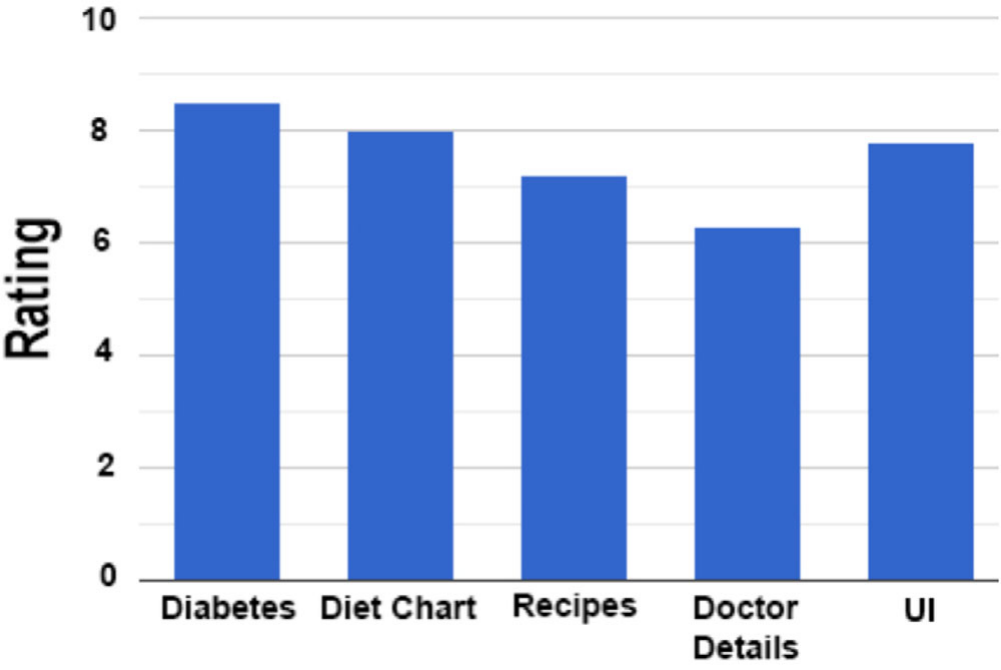
Android application review ratings

Figure 12 displays the home screen of the proposed Android mobile application created using the best classification algorithm XGBoost. Finally, a survey was conducted in which users rated the application's various features. Figure 13 illustrates the review details of the implemented Android application's survey results. Sixteen volunteers reviewed the application in total, and all of them were female. The participants rated each feature on a scale of 1 to 10, and their average was calculated. According to this figure, the diabetes prediction and daily diet chart features of the application achieved the highest ratings of 8.40 and 8, respectively.

It is worth mentioning that the RTML dataset's insulin feature has been predicted from the Pima Indian dataset by applying the XGB regression technique for all of the results discussed above. However, alternative investigations have been conducted to obtain the insulin feature of the RTML dataset, that is, the mean and median imputation of various patients’ insulin of the Pima Indian dataset. Tables 7 and  8 demonstrate various performance metrics of the machine learning models with the ADASYN technique when the RTML dataset's missing insulin features are obtained from the mean and median values of the Pima Indian dataset.

**TABLE 7:** Performance metrics of classifiers in the merged dataset (RTML insulin obtained from Pima Indian mean)

**Classifier**	**Precision**	**Recall**	**F1 Score**	**Accuracy**
AdaBoost	0.77	0.77	0.77	77%
Random Forest	0.77	0.76	0.76	76%
XGBoost	0.78	0.78	0.78	78%

**TABLE 8:** Performance metrics of classifiers in the merged dataset (RTML insulin obtained from Pima Indian median)

**Classifier**	**Precision**	**Recall**	**F1 Score**	**Accuracy**
AdaBoost	0.78	0.78	0.78	78%
Random Forest	0.76	0.76	0.76	76%
XGBoost	0.77	0.76	0.76	76%

Finally, another scenario has been considered where the insulin feature of the Pima Indian dataset has been removed to maintain consistency with the RTML dataset. Table 9 depicts various performance metrics of the merged dataset after removing the insulin feature. According to this table, the performance of all the prediction models degraded.

**TABLE 9:** Performance metrics of classifiers in the merged dataset (insulin removed from Pima Indian)

**Classifier**	**Precision**	**Recall**	**F1 Score**	**Accuracy**
AdaBoost	0.73	0.71	0.72	72%
Random Forest	0.72	0.70	0.71	71%
XGBoost	0.74	0.73	0.73	74%

Table 10 illustrates the performance comparison of the proposed automatic diabetes prediction system with similar works to the Pima Indian dataset. According to this table, the proposed XGBoost technique with ADASYN outperformed most of the existing works concerning accuracy and F1 score.

**TABLE 10:** Comparison of the proposed system with similar diabetes prediction works

**Reference**	**Classifier**	**F1 score**	**Accuracy**	**Other metrics**
[3]	Deep belief network model	0.81	N/A	Precision: 0.68 Recall: 1.0
[5]	SVM with RBF kernel		82%	
[9]	SVM	0.73	75%	Precision: 0.72 Recall: 0.75
[10]	Ensemble (XGBoost)	0.81	88.8%	Precision: 0.84 Recall: 0.79
[21]	Soft voting	0.72	79.1%	Precision: 0.73 Recall: 0.72
This work	XGBoost with ADASYN	0.81	88.5%	Precision: 0.82 Recall: 0.80

This study aims to predict diabetes mellitus automatically by employing machine learning techniques. Pima Indian dataset and a new RTML dataset comprising physical examination data from the local female patients of Bangladesh have been used. The missing insulin feature values of the RTML dataset have been predicted from the Pima Indian dataset. Our research found that the XGB regression technique accomplished the lowest RMS error in predicting insulin. The mutual information‐based feature selection algorithm indicates the glucose level, BMI, age, and insulin to be the most salient features in predicting diabetes. SMOTE and ADASYN synthetic data oversampling and hyperparameters optimization techniques have been applied. The XGBoost technique with ADASYN achieved the best performance. The LIME and SHAP explainable AI frameworks interpret the prediction provided by the ML approaches. A limitation of this study is the nonavailability of the insulin feature of the used RTML dataset. The prediction of insulin obtained from the XGB regressor and produced from the mean and median values of the Pima India dataset comprises an average deviation for classification accuracy of approximately 1.33% and 2.33%, respectively.

## 4 CONCLUSIONS

Diabetes can be a reason for reducing life expectancy and quality. Predicting this chronic disorder earlier can reduce the risk and complications of many diseases in the long run. In this paper, an automatic diabetes prediction system using various machine learning approaches has been proposed. The open‐source Pima Indian and a private dataset of female Bangladeshi patients have been used in this work. SMOTE and ADASYN preprocessing techniques have been applied to handle the issue of imbalanced class problems. This research paper reported different performance metrics, that is, precision, recall, accuracy, F1 score, and AUC for various machine learning and ensemble techniques. The XGBoost classifier achieved the best performance with 81% accuracy and an F1 score and AUC of 0.81 and 0.84, respectively, with the ADASYN approach. Next, the domain adaptation technique has been applied to demonstrate the versatility of the proposed prediction system. Finally, the best‐performed XGBoost framework has been deployed into a website and smartphone application to predict diabetes instantly. There are some future scopes of this work, for example, we recommend getting additional private data with a larger cohort of patients to get better results. Another extension of this work is combining machine learning models with fuzzy logic techniques and applying optimization approaches.

## AUTHOR CONTRIBUTIONS

Tansin Ullah Nabil: Conceptualization; Data curation; Investigation; Methodology; Software; Validation; Visualization; Writing – original draft. Sanjida Islam: Data curation; Methodology; Visualization. Riasat Khan: Project administration; Supervision; Writing – review & editing.

## CONFLICT OF INTEREST

The authors declare no conflict of interest.

## FUNDING INFORMATION

The authors received no specific funding for this work.

## Data Availability

The private dataset of female Bangladeshi patients and programming codes are available at the following link: https://github.com/tansin-nabil/Diabetes-Prediction-Using-Machine-Learning.
